# Biomarker and Companion Diagnostics—A Review of Medicinal Products Approved by the European Medicines Agency

**DOI:** 10.3389/fmed.2021.753187

**Published:** 2021-11-01

**Authors:** Laura Patricia Orellana García, Falk Ehmann, Philip A. Hines, Armin Ritzhaupt, Angela Brand

**Affiliations:** ^1^Department of International Health, Faculty of Health, Medicine and Life Sciences (FHLM), University of Maastricht, Maastricht, Netherlands; ^2^European Medicines Agency, Amsterdam, Netherlands; ^3^The United Nations University–Maastricht Economic and Social Research Institute on Innovation and Technology (UNU-MERIT), Maastricht University, Maastricht, Netherlands

**Keywords:** EPAR, IVD, IVDR, SmPC (Summary of Product Characteristics), CDx, biomarker testing

## Abstract

**Background:** An increasing number of medicines authorised in Europe recommend or require biomarker-based patient selection. For some of these the use of a companion diagnostic (CDx), a subset of *in vitro* diagnostics (IVDs), to identify patient populations eligible for a specific medicinal product may be required. The information and recommendations of use of a medicinal product for which a CDx is required is particularly important to healthcare professionals for correct patient identification.

**Methods:** We reviewed the existing information in SmPCs and European Public Assessment Reports (EPARs) of EU medicinal products approved *via* the centralised procedure at EMA where reference was made to biomarker testing, including by CDx, for patient selection.

**Results:** The results show that varying levels of detail are provided for the biomarker and the diagnostic test, including variability in where the information was presented. The overall results demonstrate transparent but sometimes heterogeneous reporting of CDx in the SmPC and EPAR.

**Conclusions:** With the introduction of the new Regulation (EU) 2017/746 on *in vitro* diagnostic medical devices, medicines regulatory authorities' will be required to be consulted during the review of CDx conformity assessment and so, there is opportunity for more consistent and transparent information on CDx to be provided in the SmPC and EPAR.

## Introduction

Healthcare has been experiencing an important change in its treatment paradigm towards personalised medicine (1). The increasing development of “omics” methods have enabled the identification of patients and the prediction of their treatment response through measuring new biological markers (biomarkers). These are critical for the success of personalised medicine, often also referred to as precision medicine or precision therapy. This approach is based on a “medical model” were biomarkers are used to ascertain the right therapeutic strategy for the right patient at the right time (2). The accurate detection of these biomarkers is key in prescribing the appropriate therapy which in turn relies on the accuracy of the *in vitro* diagnostic (IVD) tests. When IVDs are used to identify patients suitable for a specific treatment with a medicinal product they are generally referred to as companion diagnostics (CDx) (3).

Although the concept of “CDx” was first introduced in the late 1990s, when trastuzumab (Herceptin) and its corresponding assay received simultaneous regulatory approval in the USA, their regulatory context is relatively new in the European Union (EU) (4). In the EU, the regulatory assessment process for CDx is disconnected from the regulatory process of its corresponding medicinal product, and follows the regulatory requirements of *in vitro* diagnostic medical devices (5). However, with the new IVD Regulation (IVDR) (EU) 2017/746 coming into full application in May 2022, medicines regulatory authorities, including EMA assume a responsibility in reviewing the “suitability” of the CDx in relation to the corresponding medicinal product. This represents an opportunity for increasing harmonisation and consistency in the development and assessment of CDxs (6, 7). The Regulation also introduces new classification rules for IVDs, and stricter clinical evidence requirements, the ultimate goal of which is to ensure the highest level of protection and safety for patients. Furthermore, for the first time, a legal definition for CDx in Europe is set out; subject to the requirements specified in the IVDR, CDx are defined by Article 2 (7) as devices which are essential for the safe and effective use of a corresponding medicinal product to:

- “identify, before and/or during treatment, patients who are most likely to benefit from the corresponding medicinal product; or
- identify, before and/or during treatment, patients likely to be at increased risk of serious adverse reactions as a result of treatment with the corresponding medicinal product.”

The IVDR recognises that CDx are “essential for defining patients' eligibility for specific treatment with a medicinal product”. They do this by detecting treatment-specific biomarkers in order to identify subgroups of patients likely to benefit from the treatment or present a higher risk for developing adverse reactions (8).

At present there is limited information on IVDs, including CDx, contained in the Summaries of Product Characteristic (SmPC) of medicinal products authorised by the EU. The SmPC summarises the properties of medicinal products, the conditions attached to their use, and are a primary information source for healthcare professionals on how to use the medicine safely and effectively (9, 10).

The information included in SmPCs follow guidelines on what to include and where (11). If a products' indication depends on a specific genotype or expression of a gene/phenotype (e.g., biomarker-based patient selection), this information would be provided in the “Therapeutic indications” section of the SmPC (section 4.1). Information on how to use the medicinal product would be indicated at the beginning of “Posology and method of administration” (section 4.2). Information on patients with specific genotypes or phenotypes who might respond negatively is provided under the section “Special warnings and precautions for use” (section 4.4). Lastly, any relevant pharmacogenetic information from clinical studies, including specific data showing difference in the benefit/risk of between patients or patient populations would be mentioned under “Pharmacodynamic properties” (section 5.1) (11).

In addition to the SmPC, the scientific assessment of a medicinal product is summarised in the European public assessment report (EPAR), which is published for every human or veterinary medicine application that has been granted or refused a marketing authorisation *via* the centralised procedure at EMA. The EPAR includes detailed information on the evidence generated, including the clinical trials performed, submitted as part of the marketing authorisation application and how this information was assessed by EMA. The EPAR reflects the scientific conclusions of the relevant EMA committees at the end of the assessment process, providing the grounds for the opinion on whether or not to approve an application and the intended therapeutic indication(s) (12). EPARs are therefore also expected to report relevant and detailed information regarding biomarker-guided development and associated diagnostic testing (e.g., CDx).

There is an increasing number of medicinal products authorised in the EU which include certain recommendations or requirements regarding biomarkers, either for patients' selection or as a warning and precaution for clinical guidance in their SmPC (13). 15% of medicinal products evaluated by EMA in 2015 contained pharmacogenomic-related information in their label, and this is likely to increase with the technological progress expected in the field of personalised medicine (14, 15).

The information provided on biomarkers and IVDs including CDxs as part of the medicinal product labelling is important to ensure understanding and appropriate use of the medicine and CDx by healthcare professionals and patients (16). Accordingly, the wording employed acquires particular relevance too: SmPCs usually include a statement of “the use of a validated test” when referring to IVDs (17); however, at times no further information is provided to differentiate whether the diagnostic test is recommended or mandatory for the indicated use of the product. Thus, the wording currently used does not differentiate whether “the use of a validated test” refers to a CDx, or for instance, complementary diagnostics which are diagnostic tests assigned to a therapeutic class rather than a specific medicinal product (18). The information in the medicinal products labelling has been the subject of previous studies. Shekhani et al. (19) analysed pharmacogenomic data in labelling and concluded it would benefit from higher consensus across regulatory agencies and better alignment with clinical guidelines. Pignatti et al. (20) focused their analysis on the development of CDx within oncology medicines and underlined the relevance of EMA experts in assessing CDx for these medicines. As more medicines will rely on CDx in the future, and multiple tests will be accessible to detect biomarkers, it is important to ensure that information on any CDx is consistent across the instructions for use of the CDx and the type of information provided in SmPCs and EPARs to best enable the appropriate use of CDxs for a corresponding medicinal product.

This study reviewed how current information on biomarkers and associated diagnostic tests are provided in SmPCs and EPARs for medicinal products for which biomarker-based testing is recommended or mandated in view of the upcoming changes introduced by the IVDR. The study mainly focused on the type and consistency of the language when describing these diagnostic tests. Information on CDx identified as a result of this analysis were compared to the US Food and Drug Administration (FDA) approved/cleared biomarker tests (21).

## Materials and Methods

**Figure 1:**
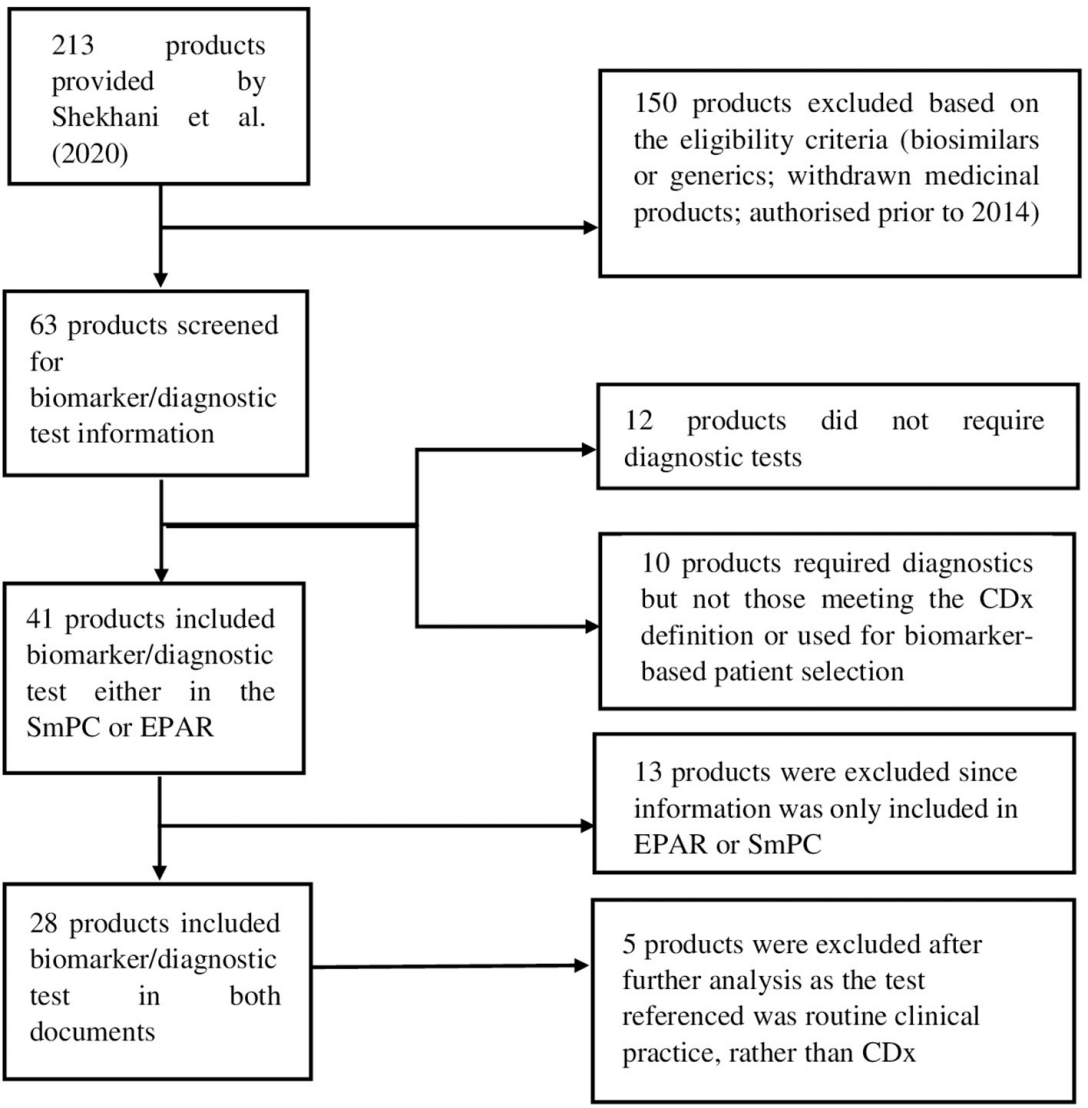
Flow diagram showing the selection process of included medicinal products and eligibility criteria.

For the purpose of this analysis, SmPC and EPAR sections were reviewed to identify the type and level of information and location included for biomarkers and associated diagnostics testing. The basis for this analysis were approved medicinal products containing pharmacogenomic labelling in the product information, identified by Shekhani et al. (19). However, for our study, only medicinal products which were granted market authorisation by the European Commission (EC) from January 2014 until June 2019, inclusive, were considered. A total of 213 medicinal products were identified in the Supplementary Tables S1–S3 provided by Shekhani et al. (19); after screening with the purpose of excluding biosimilars, generics and withdrawn medicinal products, 63 medicinal products were included for further review (Figure 1). Accordingly, the corresponding SmPCs and EPARs of these medicinal products were retrieved for detailed assessment. Biomarker-related information, and consequently information on CDx, was extracted from the four sections of the SmPC (Table 1) and two sections of EPARs (Table 2) where it is primarily reported for more detailed analysis.

**Table 1:** Sections of the SmPC where biomarker and diagnostic information are primarily located.

**Sections**	**Content**
Section 4.1	Therapeutic indications
Section 4.2	Posology and method of administration
Section 4.4	Special warnings and precautions for use
Section 5.1	Pharmacodynamic properties

**Table 2:** Sections of the EPAR where biomarker and diagnostic information are primarily located.

**Sections**	**Content**
Clinical efficacy	Dose response studies, main studies, discussion and conclusion on the clinical efficacy of the product
Benefit risk balance	Therapeutic context, favourable effects, unfavourable effects uncertainties and limitations of favourable and unfavourable effects, effects table, benefit-risk assessment and discussion

Additionally, to ensure all relevant information was captured for this analysis and not missed in other sections, the SmPCs and EPARs of the included 63 medicinal products were searched with the following terms: “assay,” “testing” or “validated test”. Then, diagnostic test-related information provided in the SmPCs and EPARs was extracted into Excel for further analysis together with information on the active substance and biomarker. Data was further categorised depending on their location in the SmPC and EPAR and on the description and level of evidence provided for the diagnostic test.

Active substances for which no information on the previous search terms or for which no specific diagnostic testing was performed in the clinical trials were excluded. Medicinal products that did not mention the need for or recommend biomarker-based patient selection were not further analysed. These medicinal products and the corresponding exclusion criteria can be found in the Supplementary Material.

The results were then grouped by biomarker and corresponding diagnostic test and whether information on a CE-marked test was provided for the IVD; in a subsequent step, it was evaluated whether the diagnostic test meets the definition of a CDx. Finally, the results were compared to the corresponding information provided in the product information of those medicinal products for which there was also a cleared or approved CDx by the FDA “*List of Cleared or Approved Companion Diagnostic Devices (In Vitro and Imaging Tools)*” (21) in June of 2020. At every stage of the research, the results were cross-checked and validated by all the authors.

## Results

After the screening process, a total of 28 medicinal products were selected for further analysis.

### Grouping of Medicinal Products Based on Reporting of Biomarkers and Diagnostic Information

In the first instance, the identified 28 medicinal products were grouped into five main categories based on the level of detail provided for the biomarker and/or the diagnostic test, i.e., was a CE-marked test available and referenced either in SmPC and/or EPAR, was a specific diagnostic test or methodology described and whether the information was described in the corresponding SmPC and/or EPAR (Table 3).

**Table 3:** Grouping of medicinal products based on reporting of biomarkers and diagnostic information.

	**Category**	**Products**
1	CE-marked test referenced in the SmPC only	Alectinib, gilteritinib, pembrolizumab
2	CE-marked test referenced in the EPAR only	Brigatinib, dacomitinib, larotrectinib, lutetium (177Lu) oxodotreotide, olaparib, rucaparib
3	CE-marked test referenced in the SmPC & EPAR	Atezolizumab, cobimetinib, durvalumab, necitumumab, nivolumab, osimertinib, talazoparib, trametinib
4	No CE-marked test referenced but description of target biomarker or methodology referenced in the SmPC and/or EPAR	Binimetinib, blinatumomab, ceritinib, encorafenib, inotuzumab, midostaurin
5	No CE-marked test referenced but reference to diagnostic/genetic test in SmPC and/or EPAR	Abacavir, allopurinol, ataluren, lumacaftor & ivacaftor, eliglustat

Of the 28 medicinal products, the majority (17, 61%) referenced the use of a CE-marked diagnostic test used during development; however, the information was not consistently found in both SmPC and EPAR. Interestingly, only 6 (21%) of the medicinal products included information on the use of a CE-marked test in the EPAR only.

The first category included medicinal products where the CE marked commercial test was used during the development and specific information on the diagnostic test was referenced in section 5.1 of the SmPC, while the same level of information was not found in the EPAR.

The second category included medicinal products where information on the use of a CE-marked diagnostic tests was provided in the EPAR only. Medicinal products in this category all had a statement requiring the use of a validated test in the SmPC section 4.2/4.4 but no reference was made whether a CE-marked test was used during development in the SmPC. The level of detail and location of the information provided differed for medicinal products in this group though, e.g., in the case of dacomitinib, section 4.2 of the SmPC included a statement that EGFR mutation status should be established prior to initiation of dacomitinib therapy and referred to section 4.4, which included the reference to use a well-validated and robust methodology to assess the EGFR mutation status of a patient.

In the case of larotrectinib and olaparib, while reference to the use of a validated test method to detect the biomarker was included in section 4.2, more elaborative information on how to conduct testing or how testing was conducted in support of the marketing authorisation application (MAA) was detailed in section 5.1.

The medicinal product containing radiolabeled Lutetium (Lu^177^) was also included in this category as it referenced the use of imaging techniques to confirm the overexpression of somatostatin receptor prior to administration in section 4.2 and included information on the specific imaging technology in the EPAR. In this particular case, the imaging technology related to radiolabeled Lutetium does not meet the criteria for a companion diagnostic, therefore it was not further considered for the purposes of this analysis.

The third category included medicinal products which referenced a CE-marked test in both the SmPC and the EPAR. However, only necitumumab did not include a statement regarding the need to use a validated test in section 4.2. While the indication for necitumumab is for patients with locally advanced or metastatic epidermal growth factor receptor (EGFR) expressing squamous non-small cell lung cancer, a direct reference to the use of a validated test was missing in the SmPC section 4.2/4.4; whereas reference to a CE-marked test was included in section 5.1. Based on the information provided in both SmPC and EPAR, one could infer that the diagnostic test referred to in section 5.1 is considered a complimentary diagnostic rather than CDx as the indication of necitumumab is for squamous non-small cell lung cancer expressing EGFR.

The fourth category included medicinal products that provided a description of the target biomarker or respective methodology in the SmPC and/or EPAR but there was no mention of a CE-marked test. While no CE-marked tests were referenced, most target biomarkers and methodologies described were related to CDxs. Each of the medicinal products in this category required the use of a validated test prior administration; this information was found in section 4.2 of the SmPC, except for encorafenib and binimetinib, which reported the requirement of a validated test in section 4.4 instead. Of note, encorafenib and binimetinib are both medicinal products which are indicated to be given together in combination.

Lastly, the remainder of the identified medicinal products were placed into group 5. All of the products in this category are indicated for therapeutic disease areas outside of oncology. These products met at least one of the screening criteria, to get to this stage of the analysis. Within this category two groups can be differentiated: medicinal products which recommended or required genetic tests for safety concerns (abacavir, allopurinol, eliglustat) and medicinal products requiring genetic testing to identify patients that can benefit from treatment (efficacy) (ataluren, lumacaftor, and ivacaftor). Both treatments for CTFR, lumacaftor and ivacaftor included reference for a “an accurate and validated genotyping method”. In the case of abacavir containing medicinal products, before initiating therapy, patients should be screened for HLA-B^*^5701 (in settings where validated screening methods are available); in the case of allopurinol (note: allopurinol was withdrawn after the analysis of the study was completed), screening for HLA-B^*^5801 should be considered before starting treatment in patient subgroups where the prevalence of this allele is known to be high; for ataluren, the presence of a non-sense mutation in the dystrophin gene should be determined by genetic testing as patients without a non-sense mutation should not receive ataluren. Eliglustat is indicated for adult patients with Gaucher disease type 1 (GD1), who are CYP2D6 poor metabolisers (PMs), intermediate metabolisers (IMs) or extensive metabolisers (EMs) and thus should be genotyped for CYP2D6 to determine the CYP2D6 metaboliser status. None of the medicinal products in this category included a reference to a particular CE-marked test. On closer inspection of the information retrieved, none of the diagnostic tests referred to meet the criteria of a CDx and thus this group of medicinal products was not further evaluated.

### Grouping of Medicinal Products by Biomarker

A number of medicinal products have been authorised in Europe based on the same biomarker, thus to compare the level and detail of the diagnostic test found in the SmPC and the EPAR, the results were further grouped by the biomarker target (Table 4).

**Table 4:** Medicinal products categorised by the biomarker target of the diagnostic test.

**Biomarker**	**Product**	**Biomarker-related indication**	**Description of diagnostic test/biomarker target**	**Location in SmPC**
ALK-positive	Alectinib	Alecensa as monotherapy is indicated for the first-line treatment of adult patients with anaplastic lymphoma kinase (ALK)-positive advanced non-small cell lung cancer (NSCLC). Alecensa as monotherapy is indicated for the treatment of adult patients with ALK-positive advanced NSCLC previously treated with crizotinib.	A validated ALK assay is necessary for the selection of ALK positive NSCLC patients. ALK-positive NSCLC status should be established prior to initiation of Alecensa therapy	4.2
	Brigatinib	Alunbrig is indicated as monotherapy for the treatment of adult patients with anaplastic lymphoma kinase (ALK)-positive advanced non-small cell lung cancer (NSCLC) previously treated with crizotinib.	ALK-positive NSCLC status should be known prior to initiation of Alunbrig therapy. A validated ALK assay is necessary for the selection of ALK positive NSCLC patients (see section 5.1). ALK-positive NSCLC status should be performed by laboratories with demonstrated proficiency in the specific technology being utilised.	4.2
	Ceritinib	Zykadia as monotherapy is indicated for the first-line treatment of adult patients with anaplastic lymphoma kinase (ALK)-positive advanced non-small cell lung cancer (NSCLC). Zykadia as monotherapy is indicated for the treatment of adult patients with anaplastic lymphoma kinase (ALK)-positive advanced non-small cell lung cancer (NSCLC) previously treated with crizotinib.	An accurate and validated ALK assay is necessary for the selection of ALK-positive NSCLC patients (see section 5.1). ALK-positive NSCLC status should be established prior to initiation of Zykadia therapy. Assessment for ALK-positive NSCLC should be performed by laboratories with demonstrated proficiency in the specific technology being utilised.	4.2
BRAF mutation	Binimetinib	Binimetinib in combination with encorafenib is indicated for the treatment of adult patients with unresectable or metastatic melanoma with a BRAF V600 mutation (see sections 4.4 and 5.1).	Before taking binimetinib in combination with encorafenib, patients must have BRAF V600 mutation confirmed by validated test. The efficacy and safety of binimetinib in combination with encorafenib have been established only in patients with tumours expressing BRAF V600E and V600K mutations.	4.4
	Cobimetinib	Cotellic is indicated for use in combination with vemurafenib for the treatment of adult patients with unresectable or metastatic melanoma with a BRAF V600 mutation (see sections 4.4 and 5.1).	Before starting this treatment, patients must have BRAF V600 mutation-positive melanoma tumour status confirmed by a validated test.	4.2
			Before taking Cotellic in combination with vemurafenib, patients must have BRAF V600 mutationpositive tumour status confirmed by a validated test.	4.4
	Encorafenib	Encorafenib in combination with binimetinib is indicated for the treatment of adult patients with unresectable or metastatic melanoma with a BRAF V600 mutation (see sections 4.4 and 5.1).	Before taking encorafenib, patients must have unresectable or metastatic melanoma with BRAF V600 mutation or metastatic colorectal cancer with BRAF V600E mutation confirmed by a validated test.	4.4
	Trametinib	Trametinib as monotherapy or in combination with dabrafenib is indicated for the treatment of adult patients with unresectable or metastatic melanoma with a BRAF V600 mutation (see sections 4.4 and 5.1).	Before taking trametinib, patients must have confirmation of BRAF V600 mutation using a validated test.	4.2
			**BRAF V600 testing** The efficacy and safety of trametinib have not been evaluated in patients whose melanoma tested negative for the BRAF V600 mutation	4.4
BRCA mutation	Olaparib	Lynparza is indicated as monotherapy for the maintenance treatment of adult patients with platinum-sensitive relapsed *BRCA*-mutated (germline and/or somatic) high grade serous epithelial ovarian, fallopian tube, or primary peritoneal cancer who are in response (complete response or partial response) to platinum-based chemotherapy.	Patients must have confirmation of a deleterious or suspected deleterious breast cancer susceptibility gene (BRCA) mutation (either germline or tumour) before Lynparza treatment is initiated. BRCA mutation status should be determined by an experienced laboratory using a validated test method (see section 5.1).	4.2
	Rucaparib	Rubraca is indicated as monotherapy treatment of adult patients with platinum sensitive, relapsed or progressive, BRCA mutated (germline and/or somatic), high-grade epithelial ovarian, fallopian tube, or primary peritoneal cancer, who have been treated with two or more prior lines of platinum based chemotherapy, and who are unable to tolerate further platinum based chemotherapy.	**Detection of BRCA mutation** There is no requirement for BRCA testing prior to using Rubraca for the maintenance treatment of adult patients with relapsed high-grade epithelial ovarian cancer (EOC), fallopian tube cancer (FTC), or primary peritoneal cancer (PPC) who are in a complete or partial response to platinum-based chemotherapy. Before taking Rubraca as treatment for relapsed or progressive EOC, FTC, or PPC, patients must have confirmation of deleterious germline or somatic mutations in the breast cancer 1 (BRCA1) or breast cancer 2 (BRCA2) gene using a validated test.	4.2
	Talazoparib	Talzenna is indicated as monotherapy for the treatment of adult patients with germline BRCA1/2-mutations, who have HER2-negative locally advanced or metastatic breast cancer. Patients should have been previously treated with an anthracycline and/or a taxane in the (neo)adjuvant, locally advanced or metastatic setting unless patients were not suitable for these treatments (see section 5.1).	Patients should be selected for the treatment of breast cancer with Talzenna based on the presence of deleterious or suspected deleterious germline BRCA mutations determined by an experienced laboratory using a validated test method.	4.2
EGFR mutation	Dacomitinib	Vizimpro, as monotherapy, is indicated for the first-line treatment of adult patients with locally advanced or metastatic non-small cell lung cancer (NSCLC) with epidermal growth factor receptor (EGFR)-activating mutations.	EGFR mutation status should be established prior to initiation of dacomitinib therapy (see section 4.4). Assessment of EGFR mutation status.	4.2
			When assessing the EGFR mutation status of a patient, it is important that a well-validated and robust methodology is chosen to avoid false negative or false positive determinations.	4.4
	Osimertinib	TAGRISSO as monotherapy is indicated for: – the first-line treatment of adult patients with locally advanced or metastatic non-small cell lung cancer (NSCLC) with activating epidermal growth factor receptor (EGFR) mutations. – the treatment of adult patients with locally advanced or metastatic EGFR T790M mutation-positive NSCLC.	When considering the use of TAGRISSO, EGFR mutation status in tumour or plasma specimens should be determined using a validated test method (see section 4.4).	4.2
			Assessment of EGFR mutation status. When considering the use of TAGRISSO as a treatment for locally advanced or metastatic NSCLC, it is important that the EGFR mutation positive status is determined. A validated test should be performed using either tumour DNA derived from a tissue sample or circulating tumour DNA (ctDNA) obtained from a plasma sample. Only robust, reliable and sensitive tests with demonstrated utility for the determination of EGFR mutation status of tumour derived DNA (from a tissue or a plasma sample) should be used. Positive determination of EGFR mutation status using either a tissue-based or plasma-based test indicates eligibility for treatment with TAGRISSO. However, if a plasma-based ctDNA test is used and the result is negative, it is advisable to follow-up with a tissue test wherever possible due to the potential for false negative results using a plasma-based test.	4.4
				4.2 4.4
	Necitumumab	Portrazza in combination with gemcitabine and cisplatin chemotherapy is indicated for the treatment of adult patients with locally advanced or metastatic epidermal growth factor receptor (EGFR) expressing squamous non-small cell lung cancer who have not received prior chemotherapy for this condition.	– Reference to a CE-marked test in SmPC & EPAR but no indication if testing is recommended or mandatory	5.1
FLT3 mutation	Gilteritinib	Xospata is indicated as monotherapy for the treatment of adult patients who have relapsed or refractory acute myeloid leukaemia (AML) with a FLT3 mutation (see sections 4.2 and 5.1).	Before taking gilteritinib, relapsed or refractory AML patients must have confirmation of FMS-like tyrosine kinase 3 (FLT3) mutation (internal tandem duplication [ITD] or tyrosine kinase domain [TKD]) using a validated test.	4.2
	Midostaurin	Rydapt is indicated: – in combination with standard daunorubicin and cytarabine induction and high-dose cytarabine consolidation chemotherapy, and for patients in complete response followed by Rydapt single agent maintenance therapy, for adult patients with newly diagnosed acute myeloid leukaemia (AML) who are FLT3 mutation-positive (see section 4.2).	Before taking midostaurin, AML patients must have confirmation of FLT3 mutation (internal tandem duplication [ITD] or tyrosine kinase domain [TKD]) using a validated test.	4.2
PD-L1	Atezolizumab	Tecentriq as monotherapy is indicated for the treatment of adult patients with locally advanced or metastatic urothelial carcinoma (UC): • who are considered cisplatin ineligible, and whose tumours have a PD-L1 expression ≥5% (see section 5.1). Tecentriq in combination with nab-paclitaxel is indicated for the treatment of adult patients with unresectable locally advanced or metastatic triple-negative breast cancer (TNBC) whose tumours have PD-L1 expression ≥1% and who have not received prior chemotherapy for metastatic disease.	**PD-L1 testing for patients with UC or TNBC** Patients with previously untreated UC and TNBC should be selected for treatment based on the tumour expression of PD-L1 confirmed by a validated test (see section 5.1).	4.2
	Durvalumab	IMFINZI as monotherapy is indicated for the treatment of locally advanced, unresectable non-small cell lung cancer (NSCLC) in adults whose tumours express PD-L1 on ≥1% of tumour cells and whose disease has not progressed following platinum-based chemoradiation therapy (see section 5.1).	**PD-L1 testing for patients with locally advanced NSCLC** Patients with locally advanced NSCLC should be evaluated for treatment based on the tumour expression of PD-L1 confirmed by a validated test (section 5.1).	4.2
	Nivolumab	Relative to nivolumab monotherapy, an increase in progression-free survival (PFS) and overall survival (OS) for the combination of nivolumab with ipilimumab is established only in patients with low tumour PD-L1 expression (see sections 4.4 and 5.1).	**Disease-specific precautions** Relative to nivolumab monotherapy, an increase in PFS for the combination of nivolumab with ipilimumab is established only in patients with low tumour PD-L1 expression. The improvement in OS was similar between nivolumab in combination with ipilimumab and nivolumab monotherapy in patients with high tumour PD-L1 expression (PD-L1 ≥ 1%). Before initiating treatment with the combination, physicians are advised to carefully evaluate the individual patient and tumour characteristics, taking into consideration the observed benefits and the toxicity of the combination relative to nivolumab monotherapy (see sections 4.8 and 5.1). Treatment of NSCLC after prior chemotherapy Factors associated with early deaths were poorer prognostic factors and/or more aggressive disease combined with low or no tumour PD-L1 expression (see section 5.1).	4.4
	Pembrolizumab	**Non-small cell lung carcinoma (NSCLC)** KEYTRUDA as monotherapy is indicated for the first-line treatment of metastatic non-small cell lung carcinoma in adults whose tumours express PD-L1 with a ≥ 50% tumour proportion score (TPS) with no EGFR or ALK positive tumour mutations. KEYTRUDA as monotherapy is indicated for the treatment of locally advanced or metastatic non-small cell lung carcinoma in adults whose tumours express PD-L1 with a ≥ 1% TPS and who have received at least one prior chemotherapy regimen. **Urothelial carcinoma** KEYTRUDA as monotherapy is indicated for the treatment of locally advanced or metastatic urothelial carcinoma in adults who are not eligible for cisplatin-containing chemotherapy and whose tumours express PD-L1 with a combined positive score (CPS) ≥ 10 (see section 5.1). **Head and neck squamous cell carcinoma (HNSCC)** KEYTRUDA, as monotherapy or in combination with platinum and 5-fluorouracil (5-FU) chemotherapy, is indicated for the first-line treatment of metastatic or unresectable recurrent head and neck squamous cell carcinoma in adults whose tumours express PD-L1 with a CPS ≥ 1 (see section 5.1). KEYTRUDA as monotherapy is indicated for the treatment of recurrent or metastatic head and neck squamous cell carcinoma in adults whose tumours express PD-L1 with a ≥50% TPS and progressing on or after platinum-containing chemotherapy (see section 5.1).	**PD-L1 testing for patients with NSCLC, urothelial carcinoma, or HNSCC** For treatment with KEYTRUDA as monotherapy, testing for PD-L1 tumour expression using a validated test is recommended to select patients with NSCLC or previously untreated urothelial carcinoma (see sections 4.1, 4.4, 4.8, and 5.1). Patients with HNSCC should be selected for treatment with KEYTRUDA as monotherapy or in combination with platinum and 5-fluorouracil (5-FU) chemotherapy based on the tumour expression of PD-L1 confirmed by a validated test (see sections 4.1, 4.4, 4.8, and 5.1). **MSI-H/dMMR testing for patients with CRC** For treatment with KEYTRUDA as monotherapy, testing for MSI-H/dMMR tumour status using a validated test is recommended to select patients with CRC (see sections 4.1 and 5.1).	4.2
			**Assessment of PD-L1 status** When assessing the PD-L1 status of the tumour, it is important that a well-validated and robust methodology is chosen to minimise false negative or false positive determinations	4.4
CD19/CD22 [B-cell precursor acute lymphoblastic leukaemia (ALL)]	Blinatumomab	BLINCYTO is indicated as monotherapy for the treatment of adults with Philadelphia chromosome negative CD19 positive relapsed or refractory B-precursor acute lymphoblastic leukaemia (ALL). BLINCYTO is indicated as monotherapy for the treatment of adults with Philadelphia chromosome negative CD19 positive B-precursor ALL in first or second complete remission with minimal residual disease (MRD) ≥0.1%. BLINCYTO is indicated as monotherapy for the treatment of paediatric patients aged 1 year or older with Philadelphia chromosome negative CD19 positive B-precursor ALL which is refractory or in relapse after receiving at least two prior therapies or in relapse after receiving prior allogeneic hematopoietic stem cell transplantation.	**MRD positive B-precursor ALL** When considering the use of BLINCYTO as a treatment for Philadelphia chromosome negative MRD positive B-precursor ALL, quantifiable MRD should be confirmed in a validated assay with minimum sensitivity of 10-4 (see section 5.1). Clinical testing of MRD, regardless of the choice of technique, should be performed by a qualified laboratory familiar with the technique, following well established technical guidelines.	4.2
	Inotuzumab	BESPONSA is indicated as monotherapy for the treatment of adults with relapsed or refractory CD22-positive B cell precursor acute lymphoblastic leukaemia (ALL). Adult patients with Philadelphia chromosome positive (Ph+) relapsed or refractory B cell precursor ALL should have failed treatment with at least 1 tyrosine kinase inhibitor (TKI).	When considering the use of BESPONSA as a treatment for relapsed or refractory B cell ALL, baseline CD22 positivity of >0% using a validated and sensitive assay is required prior to initiating treatment (see section 5.1).	4.2
NTRK	Larotrectinib	VITRAKVI as monotherapy is indicated for the treatment of adult and paediatric patients with solid tumours that display a Neurotrophic Tyrosine Receptor Kinase (*NTRK*) gene fusion.	The presence of an NTRK gene fusion in a tumour specimen should be confirmed by a validated test prior to initiation of treatment with Viktravi.	4.2

However, the level of detail provided in SmPC and EPAR differs between medicinal products that are indicated for the same biomarker-led/driven patient population as summarised below.

Brigatinib, alectinib, and ceritinib are medicinal products requiring the selection of ALK-positive NSCLC patients prior administration. All three medicinal products included reference to “a validated ALK assay is necessary” in section 4.2. Additional instruction is provided for brigatinib and ceritinib in so far that testing for ALK-positive NSCLC status should be performed by laboratories with demonstrated proficiency in the specific technology; there was no such reference in the case for ceritinib.

Medicinal products targeting BRAF V600 mutation consistently made reference to “must have” when indicating the use of a validated test to confirm BRAF V600 mutation. However, the location of the reference was not consistent: encorafenib and binimetinib included it in section 4.4, cobimetinib in section 4.2 and 4.4 whereas trametinib only reported it in section 4.2.

The reference to the use of a validated test for medicinal products targeting breast cancer gene (BRCA) mutations (rucaparib, olaparib, and talazoparib) was included in section 4.2, yet the description was not consistent: in the case of olaparib “must have” while talazoparib denoted “should be selected” when referring to BRCA mutations detection. Depending on the indications approved, rucaparib either stated the mandatory requirement of a validated test with the term “must have” (e.g., as treatment for relapsed or progressive EOC, FTC, or PPC) or explicitly specifying no BRCA testing was required (e.g., maintenance treatment of adult patients with relapsed high-grade EOC, FTC, or PPC).

Dacomitinib, osimertinib and necitumumab were identified as targeting the biomarker EGFR, and a reference to a diagnostic test was identified for all three. Both dacomitinib and osimertinib are indicated for “locally advanced or metastatic non-small cell lung cancer (NSCLC) with epidermal growth factor receptor (EGFR)-activating mutations” and a reference to the use of a validated test was included in the SmPC albeit in different sections. Dacomitinib mentioned EGFR mutation status should be established prior the initiation of the therapy in section 4.2 and reiterated the requirement of a validated test also in section 4.4. The necessity of a validated test was specified in section 4.2 for osimertinib, but additional details were provided in section 4.4 on how testing should be performed. Necitumumab's indication refers to “epidermal growth factor receptor (EGFR) expressing squamous non-small cell lung cancer” with no reference to activating mutations, thus there is no reference to the need of a validated test in section 4.2 and/or 4.4. However, reference was made to a CE-marked test used during development in section 5.1 and the EPAR, with no additional information on whether testing is recommended or mandatory.

Two medicinal products were identified requiring diagnostic tests to identify FMS-like tyrosine kinase 3 (FLT3) mutations; both medicinal products consistently specified in section 4.2 that patients “must have confirmation” of FLT3 using a validated test.

Four PD-1/PD-L1 checkpoint inhibitors were identified that target populations expressing programmed death-ligand 1 (PD-L1) The requirement (or not) of a diagnostic test before starting treatment with any of these medicinal products varied according to the approved indications. A reference in section 4.2 that patients “should be evaluated/selected” based on the confirmation of a validated test was found for atezolizumab and durvalumab. For pembrolizumab, depending on the authorised indication, the guidance found in the SmPC varied from “recommended” (e.g., to select patients with NSCLC) to “should be” (e.g., to select patients with HNSCC). No reference to mandatory testing was identified for nivolumab. Yet, information on diagnostic testing, including reference to a CE-marked test was included in SmPC 5.1. In the case of nivolumab, information was provided that no reliable cut-off could be established for PD-L1 expression to determine efficacy, implying the use of the diagnostic test as complementary rather than CDx.

Two medicinal products targeting CD19/CD22 positive B-cell precursor ALL were identified in this analysis, both included a reference to using a validated test in section 4.2. While for inotuzumab, diagnostic testing is required for baseline CD22 positivity of >0% using a validated and sensitive assay prior to initiating treatment, the reference to a validated assay for blinatumomab is in reference to quantifying presence of minimal residual disease (MRD) prior to initiating therapy. From the information provided, a CDx is not required for the use of blinatumomab in this indication.

Larotrectinib is the first so-called “histology-independent” cancer treatment that was approved in the EU requiring the confirmation of the presence of the NTRK gene fusion by a validated assay before patients can be started on the medicine. Reference to a validated test prior to initiation of treatment was included in section 4.2 with section 5.1 providing additional information on how NTRK gene fusions were identified during clinical use. In the EPAR, the different molecular tools currently available for the detection of NTRK fusions in tumour specimens were further elaborated including reference to CE-marked tests.

### Comparison to FDA-Approved Companion Diagnostics

Having identified medicinal products for which biomarker testing was referenced in the EU product information, the findings were compared to the published list of cleared companion diagnostics by the FDA (Table 5) for cross-validation. Medicinal products for which a CDx is mandatory are identified in the FDA Product Information (PI) generally in two places, as part of the indication (“as determined by an FDA-approved test”) and as part of “dosage and administration” where the link to the website for FDA-approved tests for the detection of specific biomarkers is provided (21). In addition, if a diagnostic test was used during development, this was described in the clinical studies section 14 of the US-PI, whether or not the diagnostic was considered a CDx.

**Table 5:** Comparison of medicinal products and associated companion diagnostic.

**Product**	**Reference to CE-marked CDx in EMA**	**Information found on CDx**	**FDA approved CDx**	**Reference to method/diagnostic test in section 14 of the US-PI**
Alectinib	Ventana anti-ALK (D5F3) IHC	5.1 in SmPC	Ventana anti-ALK (D5F3) IHC & FoundationOne CDx	VENTANA ALK (D5F3) CDx assay
Atezolizumab	VENTANA PD-L1 (SP142) Assay	5.1 in SmPC	VENTANA PD-L1(SP142) Assay	VENTANA PD-L1(SP142) Assay
Brigatinib	Vysis® ALK Break-Apart FISH & FoundationOne NGS	Clinical efficacy EPAR	Vysis ALK Break Apart FISH Probe Kit	Vysis® ALK Break-Apart FISH Probe Kit test
Cobimetinib	Cobas® 4800 BRAF V600 mutation test	5.1 in SmPC	Cobas 4800 BRAF V600 Mutation Test & FoundationOne CDx	Cobas® 407 4800 BRAF V600 mutation test
Dacomitinib	Qiagen therascreen EGFR Mutation Detection Kit RGS & AmoyDx EGFR Mutations Detection Kit	Clinical efficacy EPAR	Therascreen EGFR RGQ PCR Kit	Therascreen ® EGFR RGQ PCR and cobas® EGFR Mutation Test
Durvalumab	VENTANA PD-L1 (SP263) IHC assay	5.1 in SmPC	No CDx	VENTANA PD-L1 (SP263) Assay
Gilteritinib	LeukoStrat® CDx FLT3 Mutation Assay	5.1 in SmPC	LeukoStrat® CDx FLT3 Mutation Assay	LeukoStrat® CDx FLT3 Mutation Assay
Inotuzumab	Validated assay (FACS/IHC)	5.1 in SmPC	No CDx	Flow cytometry
Larotrectinib	Foundation One, FoundationOne Heme, RNA sequencing, MSK-IMPACT, Thermo Fisher Oncomine Focus, Oncoplex, Archer FusionPlex Custom, Archer FusionPlex CTL, Solid Fusion Assay, Archer FusionPlex Solid Tumour Panel; Archer Solid Tumour FusionPlex, Archer FusionPlex, Guardant360, and OmniSeq Comprehensive, OncoKids Cancer Panel, Oncomine Gene Panel, Oncopanel MDOPANELB, Sarcoma Fusion Panel, Trusight RNA Pan-Cancer Panel, ETV6 FISH and ETV6/NTRK3 FISH	5.1 in SmPC	No CDx (note: FoundationOne CDx was approved for larotrectinib after the analysis of the study was completed)	NGS or FISH
Nivolumab	PD-L1 IHC 28-8 pharmDx assay	5.1 in SmPC	PD-L1 IHC 28-8 pharmDx	PD-L1 IHC 28-8 pharmDx assay
Olaparib	Integrated BRACAnalysis assay	Clinical efficacy EPAR	Myriad myChoice® CDx, BRACAnalysis CDx & FoundationOne CDx	BRACAnalysis CDx
Osimertinib	Roche Cobas EGFR mutation test	5.1 in SmPC	FoundationOne CDx & cobas EGFR Mutation Test v2	Cobas® EGFR Mutation Test
Pembrolizumab	PD-L1 IHC 22C3 pharmDxTM Kit	5.1 in SmPC	PD-L1 IHC 22C3 pharmDxTM Kit & FoundationOne CDx	PD-L1 IHC 22C3 pharmDx kit
Rucaparib	FoundationFocus™ CDxBRCA test	Clinical efficacy EPAR	BRACAnalysis CDx, FoundationOne CDx & FoundationFocus CDx*BRCA* Assay	FoundationFocus™ CDx BRCA LOH test
Talazoparib	MYRIAD BRACAnalysis CDx®	5.1 in SmPC	BRACAnalysis CDx	BRACAnalysis CDx®
Trametinib	THxID BRAF validated assay	5.1 in SmPC	FoundationOne CDx, Oncomine Dx Target Test & THXID BRAF Kit	THxID™-BRAF assay

Generally, there was consistency between medicinal products approved by EMA and FDA that require a CDx. For inotuzumab, however, a validated test was required in the SmPC (EMA) whereas in the list of approved/cleared CDx provided by the FDA, no assay was provided. The comparison illustrated the difference between EMA and FDA when referencing the use of a recommended or required diagnostic test (i.e., CDx) in the medicinal product labelling: in the SmPC, the term “validated test” is generally used without further specifying whether such a test is to be considered a CDx or not. Of note, while in the EMA SmPC and/or EPAR, a CDx may be identified by inclusion of a reference to a validated or CE-marked test as a result of the assessment of the medicinal product, since CDx require approval by FDA, the link to the cleared list of CDx provides more transparency as to the number of CDx that are actually available for any associated medicinal product; in the EU this option is currently not available.

## Discussion

IVDs required for the selection of patients targeted by personalised medicines fall within the definition of a CDx and thus the benefit/risk of using such medicines is inevitably linked to the IVD performance for appropriate use. Consequently, appropriate information on using CDx for healthcare professionals is vital. In preparation of the new IVDR, we analysed how information on IVDs has been provided to date in the SmPCs and EPARs of medicinal products which recommend or require biomarker-based testing.

### Grouping of Medicinal Products Based on Reporting of Biomarker and Diagnostic Information

The analyses revealed that diagnostic assay/biomarker data were not always reported consistently; (i) CE-marked test referenced in the SmPC only, (ii) CE-marked test referenced in the EPAR only, (iii) CE-marked test referenced in both SmPC and EPAR, and (iv) general description of target biomarker or methodology in SmPC and/or EPAR. The information provided in the SmPC and EPAR on IVDs and CDx depends on the biomarkers' role (dependent on indication) and the information provided during the assessment for each of the medicinal products. Our analysis found that the level of detail within the SmPC and EPAR varied depending on the biomarker to be tested.

The first group of products contained more limited information than would be expected: the CE-marked tests used in the clinical trials were referenced in the SmPC only. We expected this information and more to have also been included in the EPARs, which provide more details on the assessment of the medicinal product for healthcare professionals and patients than the SmPC. On the other hand, products included in the second group did not include information on the specific tests performed in the clinical trials section of the SmPC. These products generally included information on the diagnostic test as supplementary information in the EPAR only.

This difference in reporting of diagnostic tests have recently been addressed to some extent in the updated guidance of assessment reports that ultimately make up the EPAR (22) to take note of the upcoming changes introduced by the IVDR. According to the guidance, the scientific rationale for selecting the test and its analytical/clinical strategy should be considered when editing the EPAR. These requirements exemplify the level of evidence which should be considered for when there is a requirement to use a CDx prior to initiating therapy in the EPAR. Interestingly, referencing the CE-marked test is not mentioned in the EPAR templates as relevant data to be included. The focus of the assessment from a medicinal product perspective is to provide the scientific rationale, analytical and clinical data, as key indicators of the diagnostic tests reliability, which is critical in the determination of benefit/risk of the medicinal product. In the absence of commercially available CDx, more detailed information on the performance of the CDx may be of use to healthcare institutions with in-house testing capability. However, the IVDR will also apply to healthcare institutions and corresponding laboratories that develop, manufacture, and use IVDs within their health institution (i.e., not available on an industrial scale, so called in-house IVDs). These tests were previously exempt under the IVDR. As in-house developed tests can make up a large proportion of diagnostic tests used in health institutions, often filling a gap where there is no commercially available alternative or complement CE-marked test kits, questions remain as to the impact of the IVDR on in-house developed IVDs and whether their use may be more restricted going forward if a CDx is available commercially (23, 24).

As a minimum, it should be the case that a basic level of information in terms of sensitivity/specificity or accuracy as currently requested in the updated EPAR drafting guidance is included in the SmPC and EPAR to facilitate clinical decision making. Our results reveal that even for medicinal products that require testing for the same biomarker, the level of CDx-related information, was not consistent or even missing. In view of the upcoming IVDR, and as CDx will be systematically reviewed by medicines regulators, this provides an opportunity to ensure consistent and transparent information on the key performance criteria to be included in the EPAR and thus accessible to the public. We expect that the information on CDx in the respective medicinal product's SmPC and EPAR will complement the information on CDx available *via* the European database on medical devices (EUDAMED) which is accessible for healthcare professionals and patients; together this should contribute to better safety for patients as all relevant information will be in the public domain (25).

### Grouping of Medicinal Products by Biomarker

Besides the level of evidence included, uniformity of the evidence is also relevant when providing information on IVDs in SmPCs and EPARs, as this enables downstream decision makers including health technology assessment (HTAs) bodies and Health Care trusts to take informed decisions for the implementation of an appropriate testing process. Consistent, and clear provision of information should also aid in clinical decisions and consequently patient's safety. Consistency was generally expected between medicinal products targeting the same biomarker, particularly when authorised for the same indication, while it is acknowledged that there may be some divergence in those cases were the indication evolved or there was a development in the availability of comparable biomarker tests, or their routine use, over the years. Importantly, while the indication of several medicinal products point to the same biomarker (e.g., EGFR), the wording of the indication may provide clarity whether or not prior diagnostic testing is required even if not specifically included in section 4.2 of the SmPC. For example, although necitumumab's indication refers to “epidermal growth factor receptor (EGFR) expressing squamous non-small cell lung cancer”, it does not specifically require testing for “epidermal growth factor receptor (EGFR)-activating mutations” as required for both dacomitinib and osimertinib; the latter two requiring the use of a CDx.

In addition, when the wording of the SmPC does not imply a mandatory test, it leaves room for prescribers to decide whether or not to use an IVD (17) and it could potentially lead to off-label prescribing; any consequences of such use of medicinal products may appear as part of safety reporting requirements or could be captured as part of risk management strategy. Clinical trials of personalised medicinal products are frequently conducted in patients selected by biomarker, therefore, the safety and effectiveness of the therapeutic product may be different when used in any other subpopulation.

Since there is currently no publicly available database or website that provides information on available CDx in Europe, consistency in the wording used when describing IVDs and or the need for a CDx is critical when providing guidance to healthcare professionals. In this regard, regulators play a major role in being comprehensive and consistent in their labelling terminology (26). In the SmPCs, “validated test” was regularly used to indicate the requirement to test patients for the presence (or absence) or a specific biomarker before prescribing a medicinal product. In the case of lumacaftor, the SmPC makes reference to a “validated genotyping method” to screen for a mutation in the CFTR gene. Although this may indicate that a CDx is required based on the term “validated”, the assay is in fact routine for the identification of patients suitable for treatment and therefore is not considered a CDx leaving local health trusts the liberty to implement the most suitable process. For patients and prescribers this difference may be difficult to discern.

Additional information on the testing for a specific biomarker could also help ensure HCPs and patients understand the rationale for the biomarker and subsequent CDx and prompt conversations between HCPs and patients on what may be a suitable therapy. Wang et al. (27) reviewed labels in FDA approved medicines which described the use of a biomarker, and found that the majority did not provide convincing evidence to support clinical utility of the biomarker testing recommendations. To achieve this in the EU, succinct but comprehensive biomarker data as background for CDx would be welcome (15).

### EMA-FDA Comparison

The EU centralised products associated with a CDx included in this study are discussed in comparison to the FDA approved/cleared diagnostic tests.

There was a high level of congruence between EMA and FDA for medicinal products that require a CDx, since the therapeutic indications granted by both Agencies is often identical, applications for medicinal products are usually reviewed in parallel, share the same developer and are based on the same or similar evidence and therefore the same diagnostic tests are used in the pivotal clinical trials. Of interest, since CDx requires FDA approval, this information can be accessed *via* the FDA website, and reveals if there is more than one CDx approved to be used in relation to a particular medicinal product; in the EU, currently only the assay that was used for the initial development would be referenced in EMA documents.

In the cases where a validated test was required per the EU SmPC but not according to the US product information, it is generally differences in the therapeutic indication that explain the discrepancy: in certain cases, EMA's indication is limited to a specific population (which may be biomarker based) while FDA's indication is broader. This is for example the case with inotuzumab which is authorised in the EU for patients with CD22-positive B cell precursor ALL, whereas FDA approved it for any patient with B-cell precursor ALL.

As noted already, currently in the EU there is no one location available for CDx information to be found in contrast to FDA that provides more transparency with respect to CDx. This is expected to change with the introduction of IVDR; indeed, there will be more transparency on medical devices available on the EU *via* an extended scope of the European database on medical devices (EUDAMED), the IT system developed by the European Commission and by EMA as a registration, collaborative, notification and dissemination system (open to the public) for medical devices, as well as a systematic review of CDx in conjunction with associated medicinal products which will result in more detailed information to be published as part of European public assessment reports.

## Conclusion

The overall findings of this study show that reporting of CDx by EMA is transparent but that there is room for improvement. One way to achieve this is by increasing the level of evidence in the SmPC and EPAR, including on the biomarker itself, which would provide other decision makers a more complete picture for decision making. The information should also be provided more consistently across medicinal products in the respective SmPCs and EPARs, using coherent language, unequivocally identifying whether pre-treatment testing and a given CDx is required particularly for medicinal products targeting the same biomarker-driven patient population. Although, it is important to bear in mind that divergences in reporting can be legitimate, even between products using the same biomarker and indication, as the new diagnostics and clinical practises develop.

The new IVDR offers the opportunity for EMA to increase consistency and information on biomarkers in future EPAR and SmPC guidance. The IVDR should also facilitate the identification of the CDxs associated with approved medicinal products in the EU. With the personalisation of medicines, harmonisation and consistency in the information available on CDx in medicinal products labelling will become increasingly important to help improve the understanding and appropriate use of medicine by healthcare professionals and patients.

## Data Availability Statement

The raw data supporting the conclusions of this article will be made available by the authors, without undue reservation.

## Author Contributions

LO, FE, PH, AR, and AB drafted the work, read and gave final approval of the version to be published, and provided substantial contributions to the interpretation of data for the work. All authors contributed to the article and approved the submitted version.

## Author Disclaimer

The views expressed in this article are the personal views of the authors and may not be understood or quoted as being made on behalf of or reflecting the position of the European Medicines Agency or one of its committees or working parties.

## Conflict of Interest

The authors declare that the research was conducted in the absence of any commercial or financial relationships that could be construed as a potential conflict of interest.

## Publisher's Note

All claims expressed in this article are solely those of the authors and do not necessarily represent those of their affiliated organizations, or those of the publisher, the editors and the reviewers. Any product that may be evaluated in this article, or claim that may be made by its manufacturer, is not guaranteed or endorsed by the publisher.

## Glossary

5-FU: 5-fluorouracil
ALL: Acute lymphoblastic leukaemia
ALK: Anaplastic lymphoma kinase
AML: Acute myeloid leukaemia
BRAF: v-raf murine sarcoma viral oncogene homolog B1
BRCA: Breast cancer gene
CDx: Companion diagnostics
CPS: Combined positive score
CRC: Colorectal cancer
ctDNA: Circulating tumour DNA
CTL: Comprehensive thyroid and lung
EOC: Epithelial ovarian cancer
EGFR: Epidermal growth factor receptor
Ems: Extensive metabolisers
EPAR: European Public Assessment Report
ETV6: Translocation-Ets-leukemia virus
EU: European Union
EUDAMED: European database on medical devices
FACS: Fluorescence activated cell sorting
FDA: Food and Drug Administration (US)
FLT3: FMS-like tyrosine kinase 3
FTC: Fallopian tube cancer
GD1: Gaucher disease type 1
HER2: Human epidermal growth factor receptor 2
HNSCC: Head and neck squamous cell carcinoma
HTA: Health technology assessment
IHC: Immunohistochemistry
Ims: Intermediate metabolisers
ITD: Internal tandem duplication
IVD: *In vitro* diagnostics
IVDR: IVD Regulation
FISH: Fluorescence *in situ* hybridization
LOH: Loss of heterozygosity
MAA: Marketing authorization application
MRD: Minimal residual disease
MSI-H/dMMR: Microsatellite instability-high/mismatch repair deficient
MSK-IMPACT: Integrated mutation profiling of actionable cancer targets
NGS: Next-generation sequencing
NSCLC: Non-small-cell lung cancer
NTRK: Neurotrophic Tyrosine Receptor Kinase
OS: Overall survival
PCR: Polymerase chain reaction
PD-L1: Programmed death-ligand 1
Ph+: Philadelphia chromosome positive
PFS: Progression-free survival
PI: Product information
PMs: Poor Metabolisers
PPC: Primary peritoneal cancer
SmPC: Summary of Products Characteristics
RGS: Reflection grating spectrometer
TKD: Tyrosine kinase domain
TKI: Tyrosine kinase inhibitor
TNBC: Triple-negative breast cancer
TPS: Tumour proportion score
UC: Urothelial carcinoma
