# Supplementary material for: Biomarker and Companion Diagnostics—A Review of Medicinal Products Approved by the European Medicines Agency
Source: Front Med (Lausanne). 2021 Nov 1;8:753187. doi: 10.3389/fmed.2021.753187 (PMC8591033; doi:10.3389/fmed.2021.753187)
Supplement: Supplementary file 1 [file Data_Sheet_1.docx]

**Supplementary material.**

The following tables detail the exclusion criteria of medicinal products not further analysed.

**Supplementary Table 1.** List of products (10) which did not require or recommend biomarker-based patient selection:

| Medicinal products excluded | Exclusion criteria |
| --- | --- |
| Voretigene neparvovec | Description of lab validity in EPAR was provided but validation or biomarker was not mentioned in SmPC |
| Ledipasvir 90 | Test used for patients characterisation rather than selection |
| Inotersen | The test was used in clinical trial to stratify patients but resulted on limited use |
| Irinotecan | The test was used for descriptive/exploratory purposes in the clinical trial but did not translate as a recommendation in the SmPC indication |
| Ombitasvir | The test was used for descriptive/exploratory purposes in the clinical trial but did not translate as a recommendation in the SmPC indication |
| Sofosbuvir | The test was not used for patients selection/eligibility for the treatment |
| Glecaprevir | The medicinal product did not require or recommend biomarker-based patient selection |
| Elvitegravir | A descriptive use of biomarker in clinical population was used that did not translate to SmPC |
| Atazanavir | The medicinal product did not require or recommend biomarker-based patient selection |
| Peramivir | Test used for patients characterisation rather than selection |

**Supplementary table 2.** Medicinal products excluded which did not provide information related to diagnostic tests.

| Medicinal products excluded | |
| --- | --- |
| 1 | evolocumab |
| 2 | sofosbuvir, velpatasvir; |
| 3 | brivaracetam; |
| 4 | eluxadoline; |
| 5 | sofosbuvir, velpatasvir, voxilaprevi; |
| 6 | cerliponase; |
| 7 | avatrombopag; |
| 8 | tivozanib |
| 9 | lusutrombopag; |
| 10 | patisiran |
| 11 | lesinurad |
| 12 | tacrolimus |

**Supplementary table 3.** Medicinal products excluded since information was only included in EPAR or SmPC

| Medicinal products excluded | |
| --- | --- |
| 1 | ribociclib |
| 2 | abemaciclib |
| 3 | neratinib |
| 4 | ibrutinib |
| 5 | lorlatinib |
| 6 | brexpiprazole |
| 7 | glibenclamide |
| 8 | venetoclax |
| 9 | migalastat |
| 10 | caplacizumab |
| 11 | tezacaftor, ivacaftor |
| 12 | gemtuzumab |
| 13 | elbasvir, grazoprevir |
